# Supplementary figures and images for: Microbiome analysis of Spodoptera frugiperda (Lepidoptera, Noctuidae) larvae exposed to Bacillus thuringiensis (Bt) endotoxins
Source: PeerJ. 2023 Sep 12;11:e15916. doi: 10.7717/peerj.15916 (PMC10503500; doi:10.7717/peerj.15916)

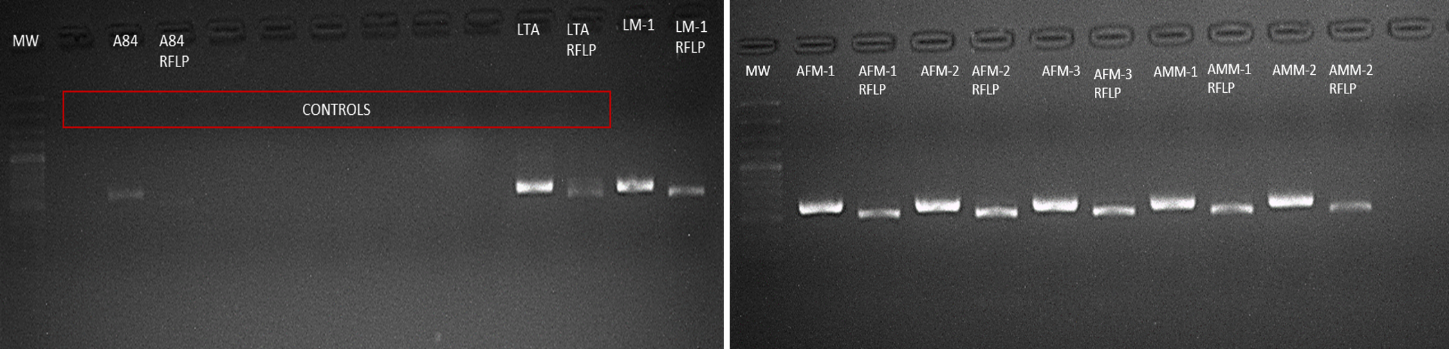

Supplement: Supplemental Information 1 — The controls correspond to: positive control A84 corresponding to the DNA previously confirmed (Higuita et al. 2021) by sequencing of the corn strain; LTA control corresponding to the total DNA of rice strain larvae where bands greater than 500bp are observed, PCR negative control and digestion negative control. The other samples were confirmed as corn strain since when performing the digestion with MSPI, bands of less than 500 bp are observed as reported (Velez-Arango 2008). The ladder used was 100 bp. [file peerj-11-15916-s001.png]

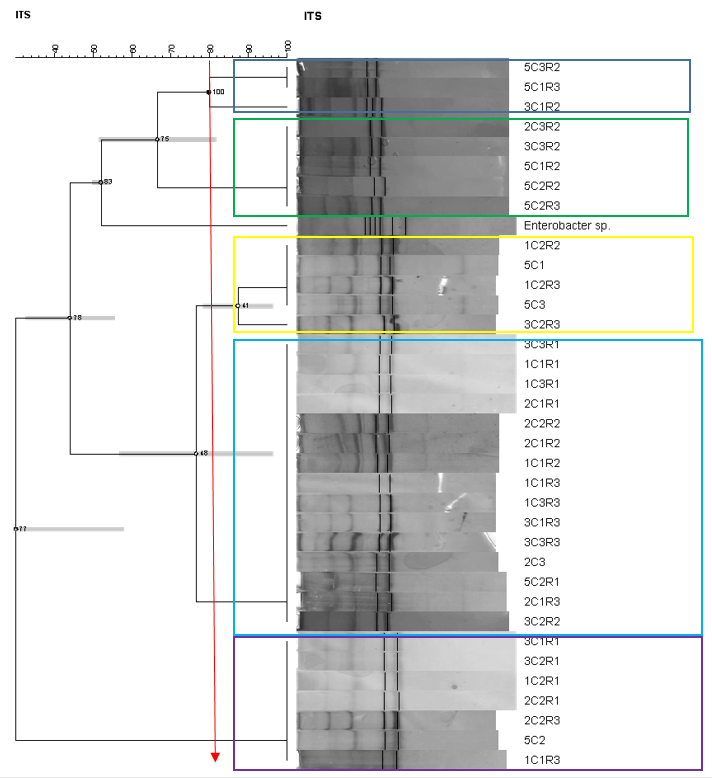

Supplement: Supplemental Information 2 — Dendogram (Pearson-Complete Linkage) obtained with GelCompar II software from the ITS region-banding patterns of the PCR fragments of the 16S rRNA genes retrieved from the total DNA from the selected colonies of intestinal samples of S. frugiperda larvae. [file peerj-11-15916-s002.png]

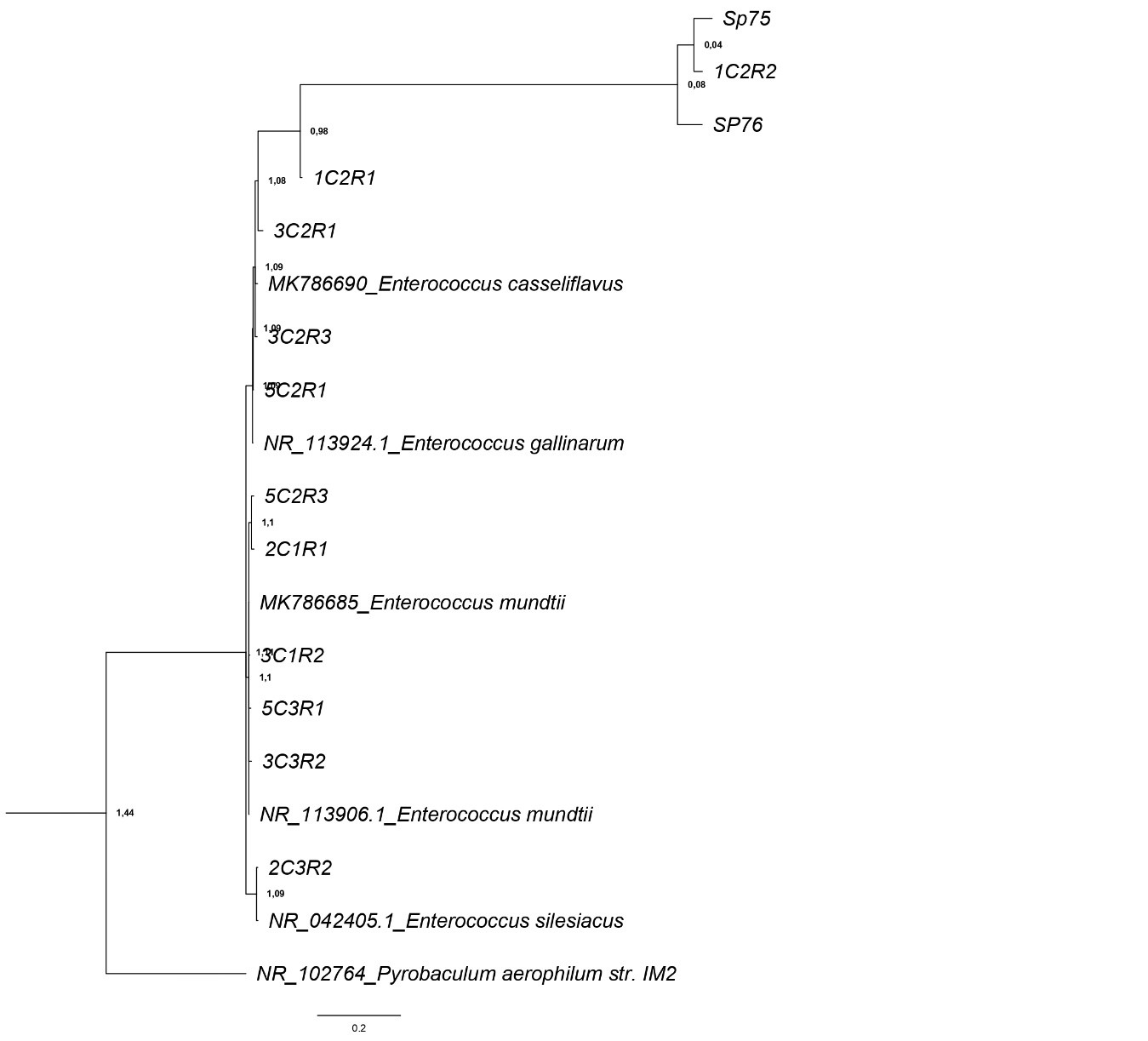

Supplement: Supplemental Information 3 — Corresponding to the bands obtained in the ITS region-banding patterns. The 16S rRNA gene sequence of the Bacterial Pyrobaculum aerophylum str. IM2 GenBank Accession Number NR_102764 was used as the outgroup. Phylogenetic tree was inferred using Bayesian analysis MrBayes (Huelsenbeck y Ronquist, 2001) under a Hasegawa-Kishino-Yano (HKY) nucleotide substitution model. The final tree was visualized using FigTree v1.3.1 (Rambaut et al., 2010). [file peerj-11-15916-s003.jpg]

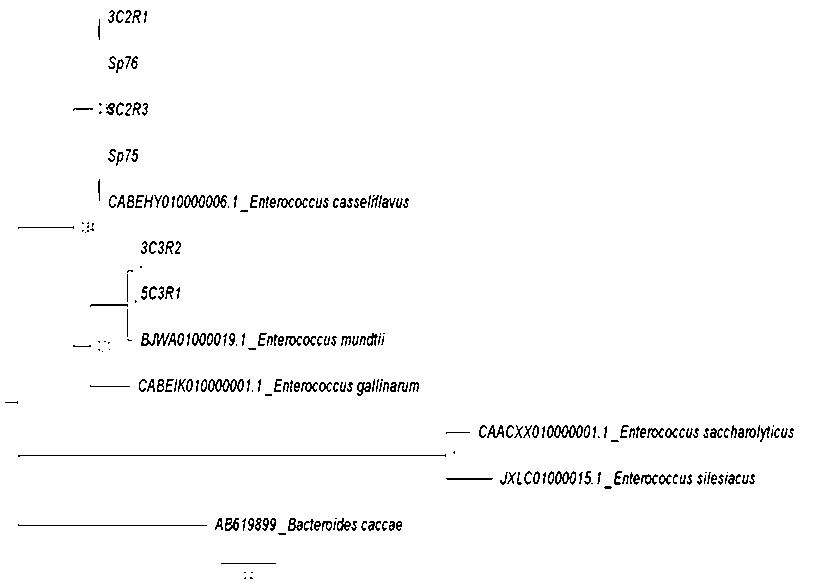

Supplement: Supplemental Information 4 — Phylogenetic tree was inferred using Bayesian analysis MrBayes (Huelsenbeck y Ronquist, 2001) under a General time reversible (GTR) nucleotide substitution model (Tavare, 1986) with gamma distribution. The appropriate model of DNA substitution was estimated with jModelTest 2.1.10 (Darriba et al. 2012) for our selected data. The trees are drawn to scale, with branch lengths measured with the maximum product of posterior probabilities. The analysis involved 19 nucleotide sequences and 12 respectively. All positions containing gaps and missing data were eliminated. [file peerj-11-15916-s004.png]

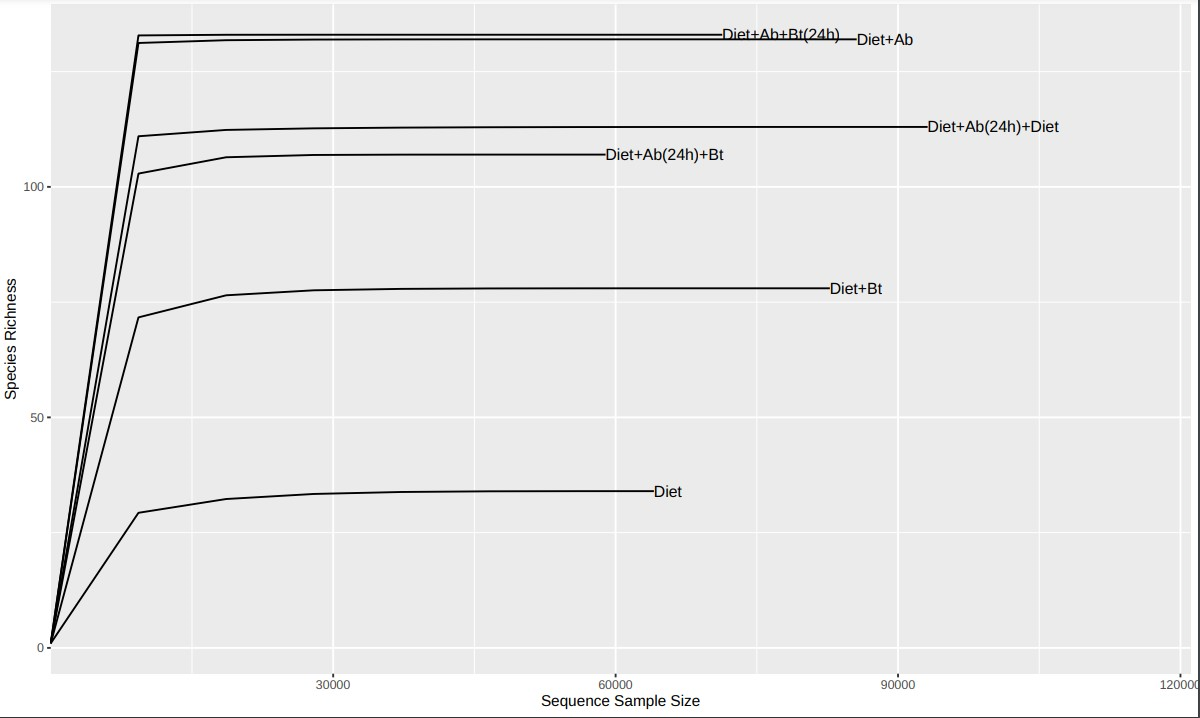

Supplement: Supplemental Information 5 [file peerj-11-15916-s005.png]

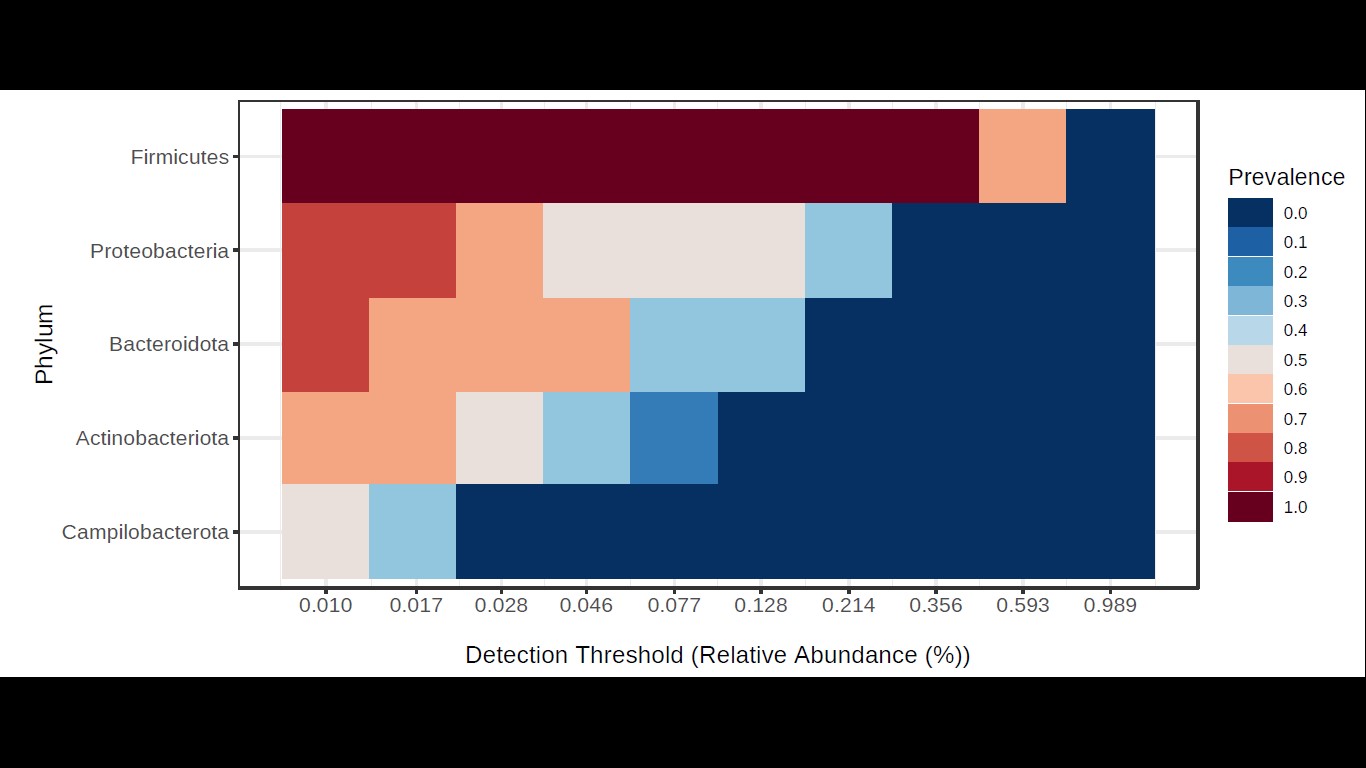

Supplement: Supplemental Information 6 — Core microbiota analysis made on phyla detected in S. frugiperda corn strain based on NGS Data. The analysis is represented in the form of a heatmap where the Y axis represents the level of prevalence of these phyla according to their relative abundance and whose range is represented on the X axis. [file peerj-11-15916-s006.jpg]
